# Association between *CYP2C19* polymorphisms and clinical outcomes in patients undergoing stent procedure for cerebral artery stenosis

**DOI:** 10.1038/s41598-021-85580-0

**Published:** 2021-03-16

**Authors:** Yan-Jiao Li, Xuan Chen, Li-Na Tao, Xin-Yuan Hu, Xin-Lu Wang, Yan-Qing Song

**Affiliations:** 1 Department of Pharmacy, The First Hospital of Jilin University, Xinmin Street 1#, Changchun, Jilin China; 2 Department of Neurosurgery, The First Hospital of Jilin University, Changchun, Jilin China; 3 Gene Diagnosis Center, The First Hospital of Jilin University, Changchun, Jilin China

**Keywords:** Clinical genetics, Genomics, Genotype

## Abstract

We investigated the effect of *CYP2C19* polymorphisms on the clinical outcomes of clopidogrel therapy in patients after stenting procedure for cerebral artery stenosis in northeast China. 568 patients performed *CYP2C19* genotype screening in the neurosurgery department of our hospital; 154 patients were finally recruited according to the inclusion and exclusion criteria, and followed-up for 6 months. Ischemic events including (1) transient ischemic attack (TIA); (2) stent thrombosis; (3) ischemic stroke; and (4) death were defined as primary clinical endpoints. The frequencies of CYP2C19*1, *2 and *3 alleles in 568 patients were 63.1%, 31.1% and 5.8%, respectively. 154 patients were classified into extensive (65 patients; 42.2%), intermediate (66 patients; 42.9%), and poor (23 patients; 14.9%) metabolizer groups. A χ^2^ test showed a significant difference in primary clinical endpoints at 6 months (P = 0.04), and a multivariate Cox regression analysis indicated that the *CYP2C19* loss-of-function (LOF) alleles associated with post-procedure prognosis. The Kaplan–Meier curve revealed that there was no significant difference in ischemic events between *2 and *3 alleles carriers. Our study verifies that CYP2C19 *2 and *3 have significant impact on the clinical outcomes of clopidogrel therapy in patients with stenting procedure for cerebral artery stenosis in China.

## Introduction

Atherosclerosis is the dominant cause of stroke, myocardial infarction, ischemic gangrene, and death, and shows a gradual progression despite risk factors controlled^1^. With the development of novel technologies, endovascular intervention has emerged as a successful alternative treatment for ischemic cerebrovascular diseases. Additionally, dual antiplatelet therapy (DAPT) represented by aspirin and clopidogrel has become the standard prescription used to reduce the occurrence of postoperative thrombus in patients with cerebrovascular stent procedures such as intracranial artery stent or carotid/vertebral artery stent^2^. A significant number of patients still experience recurrent atherothrombotic events, although clinical trials have shown the benefit of DAPT in different conditions. Previous reports have indicated that the prevalence of inadequate response to DAPT in percutaneous coronary interventions (PCIs) was 5–30%, which up to 66% in neurointerventional procedures, owing mostly to clopidogrel resistant^3–6^.

Studies have shown that clopidogrel, as a prodrug, requires hepatic cytochrome P450 (CYP) biotransformation to generate an active metabolite that can exert an antiplatelet aggregation effect. As confirmed by many studies^7–9^, CYP2C19 is the dominant enzyme affecting the metabolism of clopidogrel, which contains at least 25 variant alleles identified. Among them, the CYP2C19*1 allele is defined as functional allele, and the CYP2C19*2 and *3 as *CYP2C19* loss-of-function (LOF) alleles account for the majority of defective genotypes. According to the number of LOF alleles, individuals can be categorized into three predicted phenotypes: non-carriers, termed “extensive metabolizers” (EMs; *1/*1); patients with 1 LOF allele, termed “intermediate metabolizers” (IMs; *1/*2 and *1/*3); and patients with 2 LOF alleles, termed “poor metabolizers” (PMs; *2/*2, *2/*3, and *3/*3)^10–12^. In recent decades, numerous studies have confirmed that *CYP2C19* LOF alleles are in connection with a poor prognosis in patients with cardiovascular diseases only if they are treated with clopidogrel^13,14^. To our knowledge, very few studies have investigated this association in relation to cerebrovascular diseases, and little data have been available to guide effective antiplatelet therapy in stenting procedure.

The purpose of this study was to evaluate the effect of different *CYP2C19* genotypes on the clinical efficacy of clopidogrel therapy in patients receiving the drug after stent placement for cerebral arterial stenosis, in order to provide an evidence for individualized clopidogrel medication related to cerebrovascular diseases.

## Results

### *CYP2C19* genotype frequencies

Of the 568 patients, 338 (59.5%) carried at least one CYP LOF allele, including 81(14.3%) patients carrying two LOF alleles. The predominant genotype was CYP2C19 *1/*1 (40.5%), followed by the CYP2C19 *1/*2 genotype (37.9%). The frequency of the CYP2C19*2 (comprising *1/*2, *2/*2, and *2/*3) allele was 31.1%, and performed the same calculation for CYP2C19*3 (comprising *1/*3, *2/*3, and *3/*3) allele was 5.8% (Table 1).

**Table 1:** Genotypic and allelic frequencies and phenotypes of patients.

Gene	Genotype	Distribution, n (%)	Phenotype
	*1/*1	230 (40.5%)	EMs
	*1/*2	215 (37.9%)	IMs
*CYP2C19*	*1/*3	42 (7.4%)	IMs
	*2/*2	59 (10.4%)	PMs
	*2/*3	20 (3.5%)	PMs
	*3/*3	2 (0.3%)	PMs

EMs, extensive metabolizers; IMs, intermediate metabolizers; PMs, poor metabolizers.

### Baseline characteristics

Our study finally recruited 154 participants. According to their *CYP2C19* phenotypes, they were classified into the following 3 groups (*3/*3, n = 0): EMs, 65 patients (42.2%) (*1/*1, n = 65); IMs, 66 patients (42.9%) (*1/*2, n = 58; *1/*3, n = 8); PMs, 23 patients (14.9%) (*2/*2, n = 15; *2/*3, n = 8) (Table 2). In the cohort, there were 126 (81.8%) male and 28 (18.2%) female patients with a mean age of 61.17 years (SD = 9.03), all of whom were residents of northeast China. Of all patients, 133 (86.4%) had a degree of stenosis over 70%. The target lesion location was carotid artery in 72, vertebrobasilar system in 43, subclavian artery in 15, middle cerebral artery in 13, posterior cerebral artery in 2, and combined with two or more arteries in 9. 21 (13.6%) cases were complicated by cerebral aneurysm, among which 3 (2.0%) underwent stent-assisted intracranial aneurysm embolization simultaneously. According to the dosing regimen, 148 (96.1%) patients took aspirin combined with clopidogrel, and 6 (3.9%) received cilostazol instead of aspirin.

**Table 2:** Baseline characteristics of patients who had undergone stenting procedure for cerebral artery stenosis.

Variables	Ems (n = 65)	IMs (n = 66)	PMs (n = 23)	Total (n = 154)	P
Age, y	60.25 ± 9.13	61.27 ± 9.45	63.13 ± 7.34	61.17 ± 9.03	0.42†
Male, n (%)	54 (83.1%)	55 (83.3%)	17 (73.9%)	126 (81.8%)	0.57‡
BMI, kg/m2	23.88 (22.41–25.39)	23.88 (22.38–25.39)	23.88 (23.12–25.06)	23.88 (22.49–25.39)	0.83§
Smoking, n (%)	45 (69.2%)	38 (57.6%)	12 (52.2%)	95 (61.7%)	0.23‡
Drinking, n (%)	30 (46.2%)	31 (47.0%)	6 (26.1%)	67 (43.5%)	0.19‡
Hypertension, n (%)	42 (64.6%)	39 (59.1%)	13 (56.5%)	94 (61.0%)	0.72‡
Diabetes mellitus, n (%)	12 (18.5%)	17 (25.8%)	4 (17.4%)	33 (21.4%)	0.52‡
Coronary artery disease, n (%)	12 (18.5%)	5 (7.6%)	3 (13.0%)	20 (13.0%)	0.18‡
Complete occlusion, n (%)	7 (10.8%)	14 (21.2%)	5 (21.7%)	26 (16.9%)	0.22‡
Anterior circulation, n (%)	42 (64.6%)	34 (51.5%)	17 (73.9%)	93 (60.4%)	0.11‡
Number of stents, n (%)					0.77‡
1	54 (83.1%)	56 (84.9%)	18 (78.3%)	128 (83.1%)	
≥ 2	11 (16.9%)	10 (15.2%)	5 (21.7%)	26 (16.9%)	
Statin use, n (%)	52 (80.0%)	56 (84.9%)	21 (91.3%)	129 (83.8%)	0.43‡
CCB use, n (%)	25 (38.5%)	17 (25.8%)	8 (34.8%)	50 (32.5%)	0.29‡

^†^ P-value from one-way ANOVA test; ‡ P-value from χ^2^ test; § P-value from Kruskal–Wallis test; BMI, body mass index; CCB, calcium channel blocker.

No significant difference was found in the age (one-way ANOVA test, P = 0.42) and BMI (Mann–Whitney U test, P = 0.57) distribution among the 3 groups. There was no significant difference in vascular risk factors, such as gender, smoking history, drinking history, hypertension, diabetes mellitus, coronary artery disease, complete occlusion, anterior circulation, number of stents, statin use, and CCB use (χ^2^ test, P = 0.57, P = 0.23, P = 0.19, P = 0.72, P = 0.52, P = 0.18, P = 0.22, P = 0.11, P = 0.77, P = 0.43, and P = 0.29, respectively) (Table 2).

### Clinical endpoints

According to the results of the Cox regression model, we investigated the respective effects of the different *CYP2C19* phenotypes on clinical prognosis of participants prescribed clopidogrel after stent placement. Over the 6 months following-up, 17 (17/154) occurred endpoints, including 13 ischemic events and 6 hemorrhagic events, and 2 had complications of both an ischemic and hemorrhagic event. The primary endpoints occurred in 1 (1/69) patients in the EM group, 8 (8/66) in the IM group (2 died), and 4 (4/23) in the PM group (1 died). Using the χ^2^test, the effects of the disparate phenotypes on ischemic events were shown to be significantly different (P < 0.05), while no significant difference was found in bleeding events (P > 0.05) (Table 3).

**Table 3:** Postprocedural clinical efficacy by phenotype group.

Genotype	EMs	IMs	PMs	Total	P
Total people, n (%)	65 (42.2%)	66 (42.9%)	23 (14.9%)	154	–
Ischemic event, n (%)	1 (7.7%)	8 (61.5%)	4 (30.8%)	13	0.04
Hemorrhagic event, n (%)	3 (50.0%)	1 (66.7%)	2 (33.3%)	6	0.50

EMs, extensive metabolizers; IMs, intermediate metabolizers; PMs, poor metabolizers.

**Figure 1:**
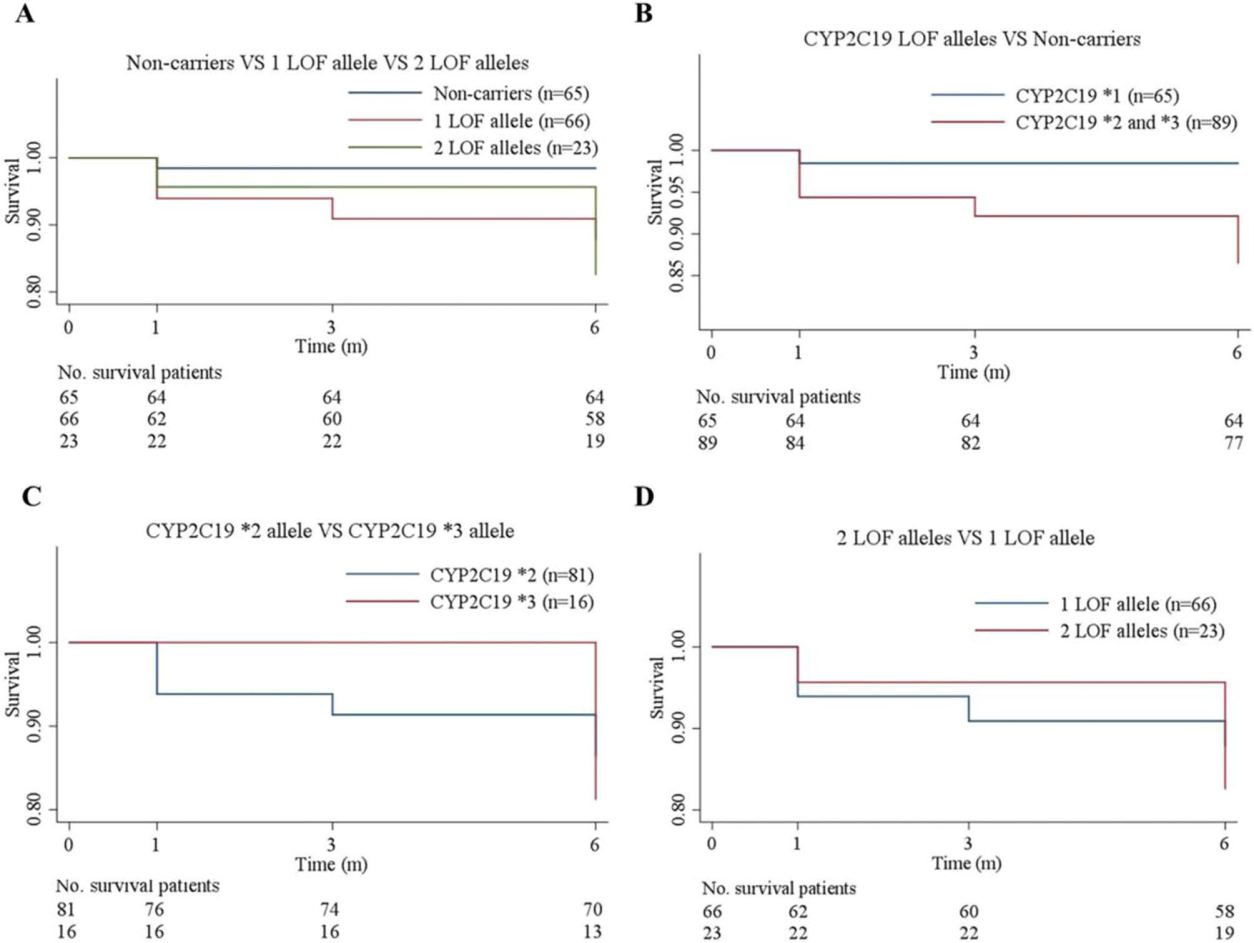
Rates of recurrence of ischemic event-free survival over 6 months of follow-up. (**A**) Patients with 1 LOF allele, including the genotypes *1/*2, and *1/*3. Patients with 2 LOF alleles, including the genotypes *2/*2 and *2/*3. The genotype of patients with non-carriers is CYP2C19 *1/*1. These curves represent the percentage of patients who survived at the primary endpoints. The figures below the curves are the numbers of patients in different groups who survived at the endpoints and remained at risk during follow-up. (**B**) Patients with *CYP2C19* LOF alleles, containing the following genotypes *1/*2, *1/*3, *2/*2, and *2/*3. (**C**) There was no difference in the cumulative survival rates between the CYP2C19*2 and CYP2C19*3 alleles. Patients with genotype *2/*3 were counted twice because *2/*3 were both included in these alleles. (**D**) There was no difference in the rates of recurrence of ischemic event-free survival between the two groups. LOF, loss-of-function. Figures were drawn using STATA 12.0 (Stata Corporation, College Station, TX, USA).

We plotted Kaplan–Meier curves to compare the 6-month recurrence of ischemic event-free survival. The curves delineated that the enrolled patients from the different phenotype groups significantly differed in terms of ischemic events (IMs vs. EMs: hazard ratio, 8.19; 95% CI of the ratio, 1.02 to 65.46; P = 0.047; and PMs vs. EMs: hazard ratio, 11.49; 95% CI of the ratio, 1.28 to 102.76; P = 0.03; Fig. 1A); and that the *CYP2C19* LOF alleles (*2 and *3) carriers were more likely to occur ischemic events than non-carriers (hazard ratio, 9.053; 95% CI of the ratio, 1.18 to 69.63; P = 0.03; Fig. 1B); In addition, we assessed whether the CYP2C19*2 and *3 alleles had the same influence on the ischemia endpoints. The results illustrated that there was no difference in the influences of CYP2C19*2 and CYP2C19*3 alleles on clinical events (hazard ratio, 1.31; 95% CI of the ratio, 0.37 to 4.69; P = 0.68; Fig. 1C). Finally, we divided the participants into two groups based on the number of LOF alleles each patient carried, as follows: 1 × *CYP2C19* LOF allele (n = 66); and 2 × *CYP2C19* LOF alleles (n = 23). The two groups have no different in the clinical endpoints (hazard ratio, 1.40; 95% CI of the ratio, 0.42 to 4.65; P = 0.58; Fig. 1D).

### Risk factors for the primary endpoints

We applied a multivariate Cox regression model to estimate independent risk factors for the primary endpoints. We included gender, age, BMI, hypertension, diabetes mellitus, coronary artery disease, smoking history, drinking history, complete occlusion, anterior circulation, number of stents, CCB use, and statin use in the multivariate Cox regression analysis. The results illustrated that the *CYP2C19* LOF alleles (*2 and *3) were risk factors for post-procedure prognosis compared with CYP2C19 *1/*1 (patients carrying one LOF allele vs. non-carriers: relative risk, 17.81; 95% CI, 1.71 to 185.39; P = 0.02; and patients carrying two LOF alleles vs. non-carriers: relative risk, 24.48; 95% CI, 1.93 to 311.30; P = 0.01) (Table 4).

**Table 4:** Risk factors for the primary endpoints.

Variables	Relative risk	95% CI	P
**Metabolizer phenotype**			
EMs	–	–	–
IMs	17.81	1.71 to 185.39	0.02
PMs	24.48	1.93 to 311.30	0.01
**Gender**			
Female	–	–	–
Male	0.41	0.07 to 2.61	0.35
**Age**			
< 60	–	–	–
60–70	0.26	0.06 to 1.17	0.08
> 70	1.12	0.23 to 5.61	0.89
BMI	0.48	0.12 to 1.98	0.31
Hypertension	1.85	0.42 to 8.22	0.42
Diabetes mellitus	0.88	0.19 to 4.09	0.87
Coronary artery disease	0.88	0.16 to 4.96	0.88
Smoking history	6.03	0.89 to 40.78	0.07
Drinking history	0.27	0.06 to 1.27	0.10
Complete occlusion	3.73	0.96 to 14.49	0.06
Anterior circulation	0.95	0.27 to 3.40	0.94
Number of stents	0.51	0.06 to 4.60	0.55
Statin use	0.48	0.11 to 1.82	0.26
CCB use	1.94	0.47 to 7.97	0.36

EMs, extensive metabolizers; IMs, intermediate metabolizers; PMs, poor metabolizers; BMI, body mass index; CCB, calcium channel blocker.

## Discussion

Some studies had reported that *CYP2C19* polymorphisms had a significant influence on clopidogrel response in patients with acute ischemic stroke, but were inconsistent in ischemic events^15,16^. In the current study, we acquired a conclusion that *CYP2C19* polymorphisms was associated with clinical outcomes of clopidogrel therapy in patients who had received stent placement for cerebral artery stenosis in China. A total of 154 participants were recruited in this study and categorized into 3 groups according to their *CYP2C19* phenotypes. A χ^2^ test showed that a significant difference was existed in endpoint events at 6 months (P = 0.04), while a multivariate Cox regression model illustrated that the *CYP2C19* LOF alleles (*2 and *3) were risk factors for prognosis after stenting procedure. Patients carrying the *CYP2C19* LOF alleles (*2 and *3) were more likely to experience ischemic events than the non-carriers, which was revealed by the Kaplan–Meier curve analysis of the primary clinical endpoints. All of these findings demonstrate that the *CYP2C19* LOF alleles (*2 and *3) are significant independent predictors for patients treated with stent procedure and clopidogrel.

In our study, among 568 patients who underwent the *CYP2C19* gene test, the frequencies of CYP2C19*1, *2, and *3 alleles were 62.1%, 32.4%, and 5.5%, respectively, similar to those that have been reported in other east Asian populations. One study reported that the frequencies of CYP2C19*2 and *3 alleles in China were 31.6% and 5.2%, respectively^17^, while frequencies of 28.6% and 8.3% were reported in another study^18^. Additionally, the Clinical Pharmacogenetics Implementation Consortium (CPIC) guidelines^19^ state that the frequencies of the CYP2C19*2 allele is ~ 15% in Caucasians and Africans, and 29–35% in Asians, while CYP2C19*3 is 2–9% in Asians, but < 1% in other populations. This indicates that the genotypic frequencies of CYP2C19*2 and *3 in Asians sum to approximately 38%, which provide a reconfirmation that the frequency of LOF alleles in Asians is higher than that in other populations. Furthermore, Asian populations are more likely to experience adverse endpoint events and more cases of low responsivity to clopidogrel because of the large Asian population.

With regard to the primary clinical endpoints, there were significant differences in the effects of different genotypes on ischemic events. This is consistent with the result of a study on the relationship between *CYP2C19* gene polymorphism and clopidogrel efficacy in patients underwent PCI. We found that the effects of the CYP2C19*2 and *3 alleles on clinical outcomes did not differ, which is in accordance with the findings of Zhu and colleagues^20^. It was further confirmed that *CYP2C19* gene screening plays a pivotal role in guiding antiplatelet therapy after stent placement. In terms of the secondary endpoints, except for the one patient with a *2/*2 genotype who died of hemorrhagic stroke during the follow-up period, no patient required hospitalization. On account of the relatively small sample size, whether the *CYP2C19* gene polymorphisms were associated with the hemorrhagic event in this one patient is uncertain.

Analysis of other factors by one-way chi-square analysis showed that these factors had no effect on the occurrence of the endpoint events. Rho and colleagues reported that hypercholesterolemia is an independent risk factor of clopidogrel resistance in patients with atherosclerotic cerebrovascular disease^21^. Lipid data were not available for many of the participants in our study, so instead we analyzed statin use as part of the baseline data; however, no significant effect of statin use was found in our study.

Several potential limitations were existed in our study. Firstly, all of the patients were recruited from our hospital and were from the northeast of China, thereby restricting the geographical region of the patient sample. Secondly, some patients with *2 and *3 alleles changed dosage regimen at the suggestion of the doctor, which meant that the number of patients with LOF alleles diminished. Although, our study remain confirms that the *CYP2C19* LOF alleles affect clinical outcomes. Therefore, larger, multi-center studies are required to verify our results. On the side, a study reported that the CYP2C19*17 allele was a risk factor for ischemic events after endovascular treatment^22^, but we were not able to obtain patient data for the CYP2C19*17 allele; thus, further *CYP2C19* gene allele testing is needed to investigate this in the future.

## Conclusion

Our results confirm that patients with the *CYP2C19* LOF alleles *2 and *3 had significantly higher numbers of ischemic events after stent placement than those with the CYP2C19 *1/*1. The *CYP2C19* genotype may be relevant to guide individualized antiplatelet treatment regimens in patients with stenting procedure for cerebral artery stenosis in China, which will improve the efficacy of clopidogrel and reduce the incidence of postoperative adverse cerebrovascular events caused by low reactivity to clopidogrel. Our findings are consistent with the results of some studies^18,22^. Our results provide a basic overview of the *CYP2C19* gene in the population of China, and verify the presence of ethnic differences in *CYP2C19* allele and genotype frequencies. At the same time, the frequency of dysfunctional genes in Asians is much higher than that in Caucasians and Africans. Based on the gene test results, a decision can be made about whether other antiplatelet drugs such as ticagrelor and prasugrel need to be used instead.

## Methods

### Study populations

**Figure 2:**
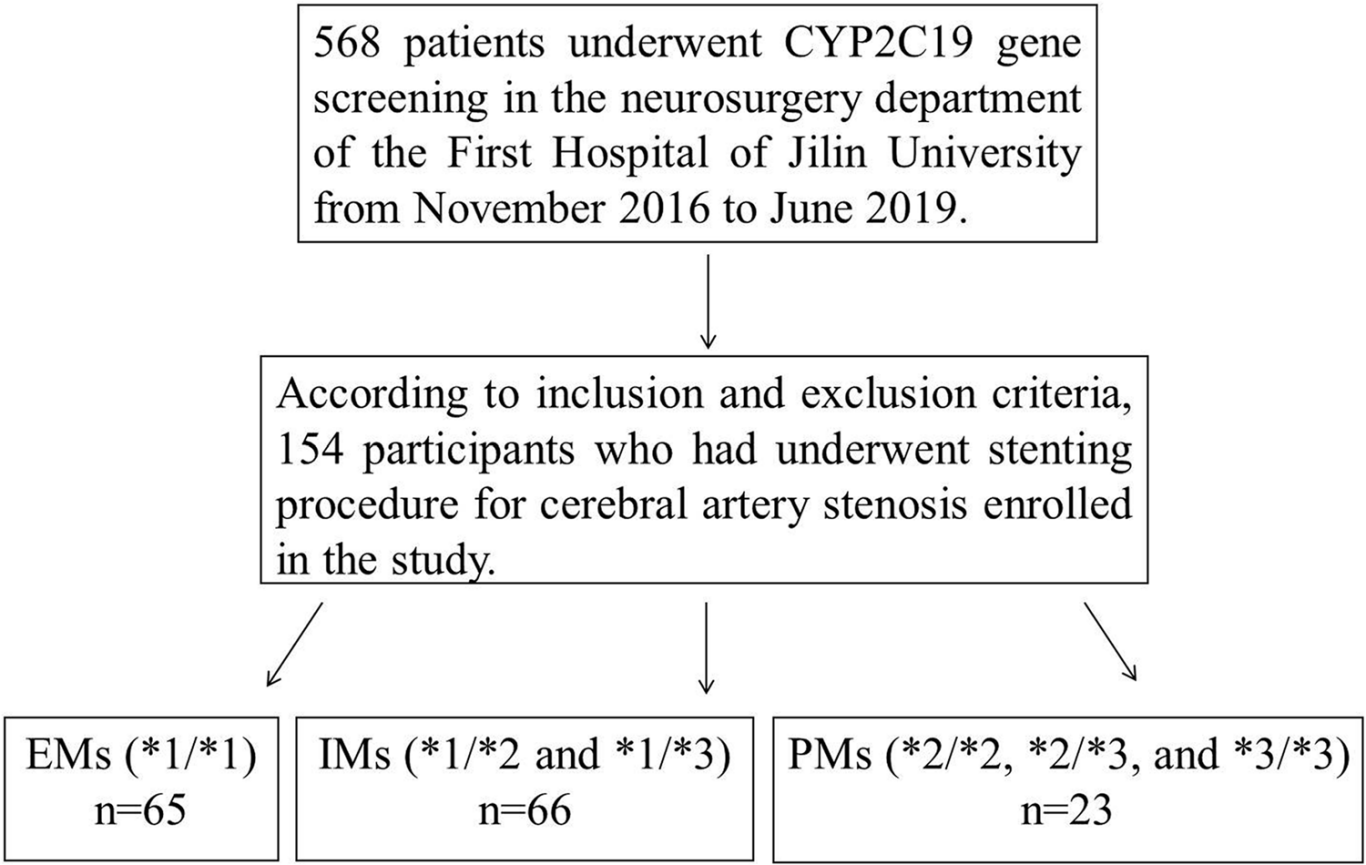
Patient screening and grouping. EMs, extensive metabolizers; IMs, intermediate metabolizers; PMs, poor metabolizers.

This study was a retrospective, single-center, cohort study and was reported in accord with the Strengthening Reporting of Observational Studies in Epidemiology statement.^23^ 568 patients who underwent *CYP2C19* gene screening and were diagnosed as cerebrovascular diseases in the neurosurgery department of our hospital were admitted to this study from November 2016 to June 2019. 154 participants were finally enrolled in the study on the basis of inclusion and exclusion criteria. According to the CPIC guidelines^19^, they were classified into three groups: group 1 (EMs; *1/*1); group 2 (IMs; *1/*2, and *1/*3); and group 3 (PMs; *2/*2, *2/*3, and *3/*3) (Fig. 2).

Inclusion criteria were as follows: (1) patients who had received cerebrovascular stenting procedures due to atherosclerotic stenosis or occlusion of the internal carotid artery (ICA), subclavian artery, vertebral artery, or intracranial arteries; (2) age older than 18 years; (3) received clopidogrel for long-term antiplatelet therapy after post-procedural DAPT; and (4) underwent *CYP2C19* gene screening.

Exclusion criteria were as follows: (1) patients who took other P2Y12 antagonists after surgery; (2) presence of a malignant tumor; (3) severe liver and kidney insufficiency; (4) allergic to clopidogrel or its metabolites; (5) coagulation dysfunction; and (6) inability to participate in return visit, so not re-examined.

The study was approved by the Medical Ethical Committee of the First Hospital of Jilin University (2016–357) and was conducted according to the Declaration of Helsinki. All participants or their authorized family members provided informed consent.

All participants’ baseline demographic information was collected as following: age; gender; body mass index (BMI; calculated as weight in kilograms divided by height in meters squared); smoking history; drinking history; diabetes mellitus; hypertension; coronary artery disease; number, size and location of stents; concomitant use of a calcium channel blocker (CCB) or statins.

### *CYP2C19* genotyping

We use a DNA-array method to screening the CYP2C19 genotype. The DNA extracted from whole blood were used the nucleic acid extracting reagent (Baio technology, shanghai, China). Genotyping for CYP2C19*1, *2 and*3 was performed using polymerase chain reaction (PCR) – restriction fragment length polymorphism. The other alleles (CYP2C19*4, *5, *6, *7, *8 and *17) were much less common and were not tested in this study. The PCR was performed according to the following protocol: 50 °C for 5 min, 94 °C for 5 min, and 35 cycles at 94 °C for 25 s, 48 °C for 40 s, and 72 °C for 30 s, followed by elongation at 72 °C for 5 min. Hybridization of the PCR products was shown with the gene probes. The data and the images were analyzed using Baio array doctor Version 2.0 (BaiO Technology Co, Ltd, Shanghai, China) software .

### Dosage and administration

According to the guidelines of intravascular interventional diagnosis and treatment of ischemic cerebrovascular diseases in China^24^, all eligible patients received aspirin and clopidogrel (at least 300 mg loading or cumulative) before the intervention, and followed by 100 mg aspirin and 75 mg clopidogrel daily after the procedure with duration of 3 months at least. Those patients who could not tolerate aspirin were prescribed 200 mg/d cilostazol.

### Endpoints and follow-up

Arrangement of follow-up was scheduled for participants at the 1st, 3rd, and 6th month after stent surgery by a specialist outpatient clinic or ward, or by telephone. We recorded whether the participants experienced a clinical endpoint within 6 months, and reviewed the postoperative outcomes of each patient by consulting the hospital case system to review information and by communicating with the doctor as necessary.

The primary clinical endpoints were ischemic events, including: (1) transient ischemic attack (TIA); (2) stent thrombosis; (3) ischemic stroke; and (4) death. TIA is defined as an acute neurological deficit that resolves within 1 h without neuroimaging evidence.

The secondary clinical endpoints were hemorrhagic events, including; (1) gastrointestinal hemorrhage; and (2) hemorrhagic stroke.

### Statistical analysis

Continuous data were described in terms of mean (SD) or the median (interquartile range, IQR) according to normal or skewed distribution. Categorical data were presented as numbers and proportions. The one-way ANOVA test was applied to compare the normal distribution continuous variables and the Kruskal–Wallis test was used to compare skewed continuous variables. The chi-square test was applied to compare nominal categorical variables and the Kruskal–Wallis test was used to compare ordinal categorical variables. Multiple Cox regression analysis was performed to identify independent risk factors associated with the primary endpoints. The Kaplan–Meier curve was plotted to compare the 6-month recurrence of ischemic event-free survival among different *CYP2C19* metabolizer groups. All shown are two-sided and P < 0.05 was defined as statistical significance. All data were analysed using SPSS version 22.0 software (IBM, Corp., Armonk, New York, USA). And figures were drawn using STATA 12.0 (Stata Corporation, College Station, TX, USA).
